# Targeted therapies in pediatric B-Cell acute lymphoblastic leukemia: mechanisms, efficacy, and future directions

**DOI:** 10.3389/fphar.2026.1654783

**Published:** 2026-01-28

**Authors:** Valeria Correa-Carranza, Guillermo Rosario-Méndez, Manuel Castillejos-López, Juan Luis Chávez-Pacheco, Cesar Galván-Díaz, Luz María Torres-Espíndola

**Affiliations:** 1 Laboratorio de Farmacología, Instituto Nacional de Pediatría, Ciudad de México, Mexico; 2 Maestría en Ciencias de la Salud, Escuela Superior de Medicina. Instituto Politécnico Nacional, Ciudad de México, México; 3 Maestría en Ciencias Químico-Biológicas, Escuela Nacional de Ciencias Biológicas, Instituto Politécnico Nacional, Ciudad de México, México; 4 Laboratorio de Investigación en Epidemiología y Enfermedades Infecciosas, Instituto Nacional de Enfermedades Respiratorias “Ismael Cosío Villegas, Ciudad de México, México; 5 Servicio de Oncología, Instituto Nacional de Pediatría, Ciudad de México, México

**Keywords:** cancer, immunotherapy, leukemia, Pediatrics, proteasome inhibitors, protein kinase inhibitors, targeted therapy

## Abstract

**Background:**

Acute lymphoblastic leukemia (ALL) is the most common hematologic malignancy in children and is characterized by rapid progression and, in some cases, a high risk of relapse. Targeted therapies have revolutionized treatment with greater specificity, reduced systemic toxicity and a better prognosis.

**Objective:**

This review provides a comprehensive analysis of current targeted therapies for pediatric B-cell ALL, focusing on their mechanisms of action, efficacy, safety profiles, advantages, and remaining challenges.

**Methods:**

A systematic review of clinical trials published over the past 15 years was conducted. The analyzed therapies include monoclonal antibodies, antibody‒drug conjugates, tyrosine kinase inhibitors, proteasome inhibitors, and chimeric antigen–receptor T-cell (CAR-T cell) immunotherapy.

**Results:**

Targeted therapies improved progression-free survival and overall response rates, particularly in patients with relapsed/refractory ALL. CD19-directed CAR-T-cell therapy and bispecific antibodies (e.g., blinatumomab) have demonstrated high remission rates in early-phase clinical trials. Additionally, BCR-ABL1-positive ALL patients show benefit from tyrosine kinase inhibitors when combined with chemotherapy.

**Conclusion:**

Targeted therapies represent a paradigm shift in ALL treatments, enabling more personalized and effective strategies. Their integration into standard protocols, especially for high-risk and relapsed patients, is crucial to enhancing long-term outcomes.

**Systematic Review Registration:**

https://www.crd.york.ac.uk/PROSPERO/view/CRD420251110522, identifier CRD420251110522

## 1 Introduction

Leukemia is the most common type of cancer in children worldwide. Acute lymphoblastic leukemia (ALL) is the most prevalent subtype, followed by acute myeloid leukemia. In Mexico, ALL accounts for 85.35% of childhood leukemia cases, making it the most common leukemia among children (Secretaría de Salud, 2019). The highest incidence is observed in children aged 1–4 years, with 81 cases per million each year. Among Hispanic children, the rate is 48 cases per million annually (Malard and Mohty, 2020; National Cancer Institute, 2024). Approximately 85% of pediatric ALL cases are of the B-cell type (Bhojwani et al., 2015). This underscores the need to review targeted therapies for this subtype, particularly their effectiveness and safety. The standard treatment for ALL consists of three phases: induction, consolidation, and intensification/maintenance, along with preventive treatment for the central nervous system (Malard and Mohty, 2020). Regimens such as the Berlin–Frankfurt–Münster (BFM) protocol incorporate medications such as prednisone, vincristine, daunorubicin, and asparaginase. These treatments have proven effective in Latin America, with overall survival (OS) rates ranging between 60% and 70%, and relapse rates of 66.1% (Castro-Arechaga et al., 2018). A comprehensive study of the BFM regimen reported an OS rate of 82.6%, a relapse rate of 17.3%, and an 82.6% negativity rate for minimal residual disease (MRD) by day 15. Common side effects included infections (31.4%), allergic reactions to asparaginase (20.9%), and rarer issues such as neurological problems (5.01%) and pancreatitis (1.04%) (Campbell et al., 2023). Further research is necessary to evaluate the quality of life of patients in Latin America. Despite advances in pediatric cancer treatments, survival rates can vary due to socioeconomic factors. High-income countries often achieve survival rates exceeding 80%, whereas many low-income regions, including much of Latin America, report rates as low as 30% (Organización Panamericana de la Salud and Organización Mundial de la Salud, 2025). Traditional chemotherapy is effective but can cause severe side effects, and higher doses are often limited in more challenging cases (Pui, 2020). Targeted therapies, which include innovative medications and treatments, present emerging therapeutic opportunities. Some trials indicate an 80% chance of event-free survival and an OS of more than 90%, with lower relapse rates (Pui, 2020). Recent advancements in understanding ALL and identifying patient risk factors have facilitated the development of targeted therapies. Research into B-cell genetics and surface proteins has led to novel treatments, such as tyrosine kinase inhibitors, proteasome inhibitors, chimeric antigen receptor T-cell (CAR-T cell) therapies, and monoclonal antibodies that leverage the body’s immune system to fight against cancer (Micallef et al., 2022). However, high costs hinder access to targeted therapies for many patients in Latin America, which restricts participation in clinical trials and the routine use of these treatments. In 2023, an expert panel across Latin America recommended therapies like rituximab, blinatumomab, and inotuzumab ozogamicin. Nonetheless, access remains largely confined to wealthier areas or through industry-funded trials (Basquiera et al., 2023). In Mexico, blinatumomab was approved in 2018 for the treatment of pediatric patients with Ph-negative relapsed/refractory ALL (Farma, 2018), and recently, it was approved in 2024 as consolidation therapy in ALL, making it the first immunotherapy for ALL in the country, although local trial data are lacking. Inotuzumab ozogamicin (targeting CD22) and dasatinib for pediatric Ph + ALL are also approved but are restricted to later lines of treatment (Comisión Federal para la Protección contra Riesgos Sanitarios, 2024). While these therapies may have fewer side effects, long-term studies are needed to determine their superiority over traditional chemotherapy for children. This review aims to analyze targeted therapies for pediatric B-cell ALL, focusing on their mechanisms, effectiveness, safety, benefits, and challenges.

## 2 Methodology

The Population, Intervention, Comparison and Outcome (PICO) framework was applied to develop a targeted investigation. This approach enabled us to formulate the following research question: Is there a difference in efficacy, safety, and quality of life outcomes between targeted therapies (administered as monotherapy or in combination with chemotherapy) and standard chemotherapy alone in pediatric patients with B-cell acute lymphoblastic leukemia? To answer this question, we designed a systematic review with a search algorithm that incorporated MeSH (Medical Subject Headings) terms: ((Precursor Cell Lymphoblastic Leukemia-Lymphoma) AND ((Pediatrics) OR (Child)) AND ((Protein Kinase Inhibitors [Pharmacological Action) OR (Protein Kinase Inhibitors) OR (“Proteasome Inhibitors”) OR (“Antineoplastic Agents, Immunological”) OR (bispecific antibody) OR (monoclonal antibody) OR (“CAR T cells”) AND (Clinical Trial [Publication Type])) AND (chemotherapy)). The systematic search was carried out on a single date, October 23, 2024, applying the algorithm in the EMBASE, MEDLINE, Cochrane and LILACS databases. No additional filters were applied to the algorithm, so articles in any language and publication date were considered; grey literature was not included. The initial search yielded 350 potentially relevant records.

Strict selection criteria were established. To be included, studies had to focus on pediatric patients (≤18 years) with a confirmed diagnosis of B-cell ALL. Studies evaluating targeted therapies, defined as agents designed to act on specific molecular targets or cell surface antigens (including protein kinase inhibitors, monoclonal antibodies, bispecific antibodies, and CAR-T cells), were included. Therapies administered in combination with standard chemotherapy regimens were considered, as were those used as monotherapy or replacement therapy in relapsed or refractory disease, provided that the comparison group was conventional chemotherapy. And report at least one outcome related to efficacy, safety, or quality of life. We excluded studies involving adult populations, T-cell ALL patients, preclinical investigations, and publications that were not clinical trial reports.

The article selection process was conducted in two phases. In the first phase, two independent authors reviewed the titles and abstracts of the 350 identified records, selecting those that met the inclusion criteria. Although the kappa coefficient was not calculated due to the low number of discrepancies, a standardized consensus process was applied to ensure inter-observer reliability. In the second phase, these same researchers evaluated the full texts of the preselected articles in detail. To ensure consistency in data extraction, we used standardized tables that captured key characteristics, such as authors, year of publication, study design, population demographics, details of interventions, comparator groups, and reported outcomes. In cases of disagreement between the investigators, a third evaluator resolved the discrepancy.

The risk of bias in the included studies was assessed according to their methodological design. Randomized controlled trials were assessed using the Cochrane Risk of Bias 2 (RoB 2) tool, which considers five domains: randomization, deviations from the planned intervention, missing outcome data, outcome measurement, and selection of the reported outcome. Each domain is classified as low, concerning, or high risk. For non-randomized studies, the analysis was performed using the Risk of Bias in Non-Randomized Interventions I (ROBINS-I) tool, which considers six domains and classifies them as low, moderate, high, or critical risk. The quality of the evidence was assessed using the Grading of Recommendations, Assessment, Development and Evaluation (GRADE) system, which classifies confidence in treatment effect estimates into four levels: high (high confidence that the estimated effect is close to the true effect), moderate (probable closeness with the possibility of substantial difference), low (limited confidence with possible significant difference), and very low (very limited confidence with probable substantial difference). This approach helped us determine levels of confidence in the reported results, facilitating the interpretation of the evidence for developing recommendations (Fisterra, 2024).

**FIGURE 1:**
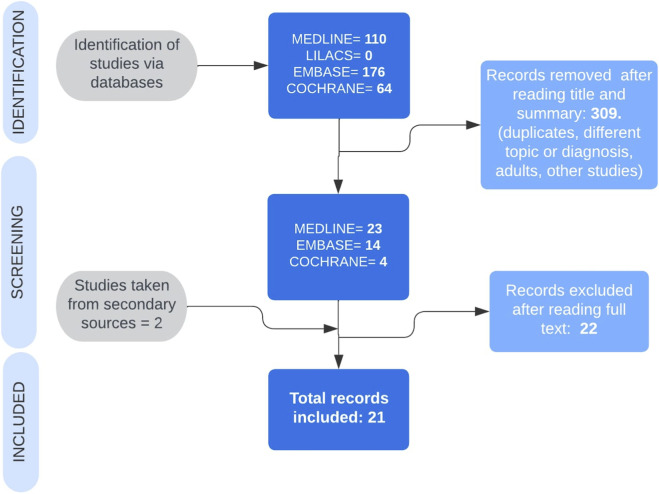
Search process and collection of articles. Created in BioRender. CORREA, V. (2026) https://BioRender.com/nmbkbkx.

From the initial total of 350 records, 309 were excluded during the initial review of titles and abstracts because they did not meet the inclusion criteria (studies involving adult populations, book chapters, nonclinical research, and topics outside the scope of the review). During the full-text assessment, we excluded an additional 22 documents for not fully meeting the inclusion criteria, resulting in the final inclusion of 21 primary studies for our analysis. This rigorous methodological process ensured that our review was based on the most relevant and reliable evidence available on the topic. Figure 1 outlines the process used for the systematic identification and selection of relevant literature.

This systematic review was previously registered in the PROSPERO database (registration: CRD420251110522), and was conducted in strict accordance with the recommendations set out in the PRISMA 2020 guidelines.

### 2.1 Data analysis

The extracted data were systematically organized into tabular formats and subjected to descriptive statistical analysis. Comparative analyses of the study results were conducted, focusing on key outcome measures: efficacy, disease-free survival, complete response rate, safety, incidence of serious adverse events, and quality of life as assessed by validated psychometric scales. Furthermore, trends and patterns within the data were identified to evaluate the consistency and robustness of the accumulated evidence.

## 3 Results

### 3.1 Protein kinase inhibitors in pediatric oncology: general overview

**FIGURE 2:**
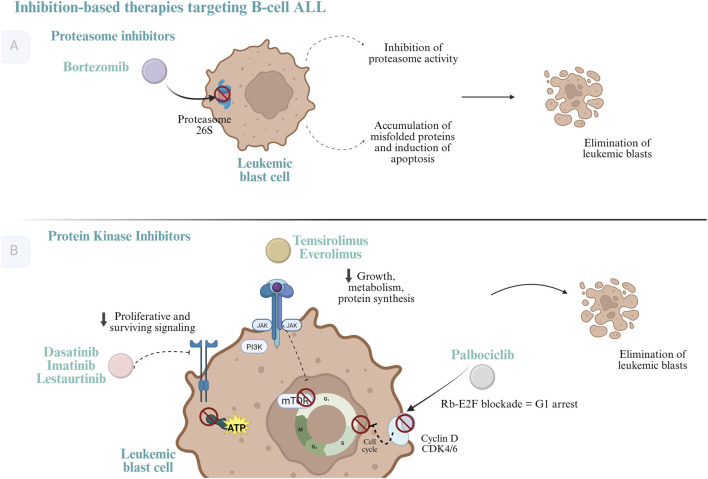
Inhibition-based therapies targeting B-cell acute lymphoblastic leukemia. **(A)** Proteasome inhibitors. Bortezomib block the activity of the 26S proteasome in leukemic blast cells, leading to impaired degradation of regulatory and misfolded proteins. This results in disruption of cell-cycle control, induction of apoptotic signaling pathways, and ultimately the elimination of leukemic blasts. **(B)** Protein kinase and cell-cycle inhibitors. Small-molecule inhibitors targeting key signaling pathways involved in leukemic cell proliferation and survival include tyrosine kinase inhibitors (e.g., dasatinib, imatinib, and lestaurtinib), inhibitors of the PI3K–AKT–mTOR pathway (e.g., temsirolimus and everolimus), and CDK4/6 inhibitors (e.g., palbociclib). By suppressing oncogenic signaling, inhibiting cellular growth and protein synthesis, and inducing cell-cycle arrest through Rb–E2F blockade, these agents promote growth inhibition and apoptotic cell death, ultimately resulting in the elimination of leukemic blasts. Created in BioRender. CORREA, V. (2026) https://BioRender.com/nmbkbkx.

Protein kinase inhibitors have revolutionized cancer treatment by targeting specific molecular alterations related to tumor pathophysiology. These enzymes regulate essential cellular processes -such as proliferation, apoptosis, inflammation, and metabolism-through substrate phosphorylation (Attwood et al., 2021). The aberrant activation of these genes, often resulting from mutations or genetic translocations, is well documented as a key oncogenic event in various solid and hematologic malignancies (Stransky et al., 2014). Advances in understanding these mechanisms have led to the development of targeted therapies that selectively inhibit kinase activity, typically by occupying the catalytic site, thereby reducing enzymatic function and halting tumor progression (Sravani and Duganath, 2012). This review identified six inhibitors with clinical or experimental applications in pediatric oncology, namely, imatinib, dasatinib, lestaurtinib, palbociclib, temsirolimus, and everolimus. Their mechanisms of action are depicted in Figure 2.

#### 3.1.1 Imatinib and dasatinib: BCR-ABL inhibitors

Imatinib was the first tyrosine kinase inhibitor approved by the FDA in 2001 for treating chronic myeloid leukemia (CML) in adults. It works by inhibiting the BCR-ABL fusion protein, the product of the t (9; 22) translocation that creates the Philadelphia chromosome (Ph) Positive (Cohen, et al., 2021). This alteration is also found in a subset of pediatric B-cell ALL patients, with a frequency of approximately 3%–4% (Brivio, et al., 2022). It is associated with poor prognosis and high relapse risk.

Clinical studies have shown that adding imatinib to standard chemotherapy regimens—during or after induction—significantly improves event-free survival in standard-risk and high-risk patients. No significant differences in grade III-IV adverse events were observed between the treated and untreated groups; the most common toxicity was infection associated with myelosuppression (Biondi, et al., 2018; Rives et al., 2011).

A phase III trial involving 156 pediatric CML patients reported an 18-month event-free survival rate of 97% (95% CI: 94.2%–99.9%), with favorable hematologic, cytogenetic, and molecular responses. In this study, 30% of patients experienced grade 3–4 hematologic toxicity; gastrointestinal (38%) and musculoskeletal (36%) were the most common nonhematologic toxicities (Suttorp, et al., 2018).

Dasatinib, a second-generation inhibitor initially approved in 2006 for adults with CML and later in 2018 for pediatric use, has shown superior clinical outcomes compared with imatinib in patients with Ph-positive B-ALL (Cohen, et al., 2021). Randomized clinical trials have demonstrated improved event-free survival and higher remission rates with the use of dasatinib; while adverse effects are like those of imatinib, hematologic toxicities have been reported, including grade ≥3 neutropenia (81%), thrombocytopenia (67%), and anemia (58%) (Shen, et al., 2020). Additionally, its combination with chemotherapy has shown efficacy in patients with relapsed or refractory neuroblastoma with c-Myc amplification, prolonging progression-free survival. In addition, one treatment-related death occurred, underscoring the need for close monitoring (Corbacioglu, et al., 2024).

#### 3.1.2 Lestaurtinib: FLT3 inhibitor with limited efficacy

Lestaurdinib, an FLT3 tyrosine kinase inhibitor, has been evaluated in ALL and AML, in which this kinase is overexpressed (Roskoski, 2016). It induces apoptosis in transformed hematopoietic cells. However, its clinical efficacy in pediatric populations has been limited. In a randomized trial of high-risk B-ALL patients, adding lestaurtinib to chemotherapy did not improve survival rates or reduce the 3-year relapse incidence. The most common toxicities were elevated transaminase levels (37%–51%), neutropenia (58%), and diarrhea (34%, p = 0.04) (Brown, et al., 2021a).

Similarly, a phase I trial in neuroblastoma patients showed minimal clinical response, with most patients discontinuing treatment owing to progression. The dose-limiting toxicity was hepatotoxicity, which was observed at doses of 70 mg/m^2^/day or higher (Minturn et al., 2011).

#### 3.1.3 Palbociclib: cell cycle inhibitor

Palbociclib, a cyclin-dependent kinase 4/6 (CDK4/6) inhibitor approved in 2016 for ER+/HER2− breast cancer, has recently been explored in pediatric cancer. It halts cell cycle progression from G1 phase to the S phase, inducing tumor cell senescence (Raetz et al., 2023; Morrison, et al., 2024). In a phase II study in pediatric B-ALL patients, combining palbociclib with chemotherapy resulted in remission in three of seven patients; however, this study also included young adults. As monotherapy, it showed grade 3–4 hematologic toxicities, primarily neutropenia and thrombocytopenia (Raetz et al., 2023). As monotherapy in patients with neuroblastoma, it showed limited activity: two patients with CDK4 amplification achieved stable disease (Macy, et al., 2024).

#### 3.1.4 Temsirolimus and everolimus: mTOR inhibitors

Temsirolimus, an mTOR complex inhibitor, downregulates hypoxia-inducible factor 1-alpha (HIF-1α), reducing VEGF production and limiting tumor angiogenesis (Chen, et al., 2019). In relapsed B-ALL, temsirolimus combined with reinduction chemotherapy led to three cases of complete remission with negative minimal residual disease in a cohort of 16 patients. The most reported toxicities associated with temsirolimus were febrile neutropenia (67%) and mucositis (27%) (Rheingold, et al., 2017).

However, a randomized trial for refractory neuroblastoma showed lower efficacy in terms of progression-free survival (PFS) and OS than did dinutuximab, which was selected for continued clinical development. Sometimes, dose reduction was necessary due to hematologic and infusion-related adverse events (Mody et al., 2017).

Finally, everolimus, another mTOR inhibitor, has been approved for treating pediatric tumors such as subependymal giant cell astrocytoma and renal angiomyolipoma in the context of tuberous sclerosis (Davies, et al., 2017). In B-ALL patients, everolimus reduced blast counts in 44% of patients within the first 3 days of treatment. The most common adverse effects included febrile neutropenia, infections, elevation of transaminases, hyperbilirubinemia and hypophosphatemia (Place, et al., 2018).

In pediatric patients with low-grade glioma, a 6-month progression-free survival rate of 67.4% was reported, with a median overall survival of 11.1 months. Hypertriglyceridemia (16.9%) and gastrointestinal toxicity (15.4%) were the most common adverse effects (Haas-Kogan, et al., 2024).

These findings highlight the emerging and diverse role of kinase inhibitors in pediatric oncology, underscoring the need for better molecular characterization and rigorous evaluation of their efficacy and safety through controlled clinical trials (Table 1).

**TABLE 1:** Outcomes of clinical trials in pediatric patients with B-cell acute lymphoblastic leukemia treated with protein kinase and proteasome inhibitors.

Author (year)	Study	N	Median age (y)	Intervention	Efficacy	Safety	RoB2/ROBINS-I	GRADE
Protein kinase inhibitor therapy								
Hunger (2023)	Phase II, multicenter	104	9.4	Dasatinib 60 mg/m^2^ + CT	EFS 3 years: 65.5% [95% CI, 55.5-73.7], OS 3 years: 91.5% [95% CI, 84.2-95.5] OS 5 years: 81.7% [95% CI, 72.8-87.9]	Neutropenic fever (89%), septicemia (25%)	Moderate risk	Moderate
Shen et al. (2020)	Phase III, multicenter	189	7.8	Dasatinib vs. imatinib + CT	EFS 4 years: 71% [95% CI, 56.2-89.6] vs. 48.9% [95% CI, 32-74.5] OS: 69.2% [95% CI, 55.6-86.2] vs. 88.4% [95% CI, 81.3-96.1]	Similar events in both groups	Moderate risk	High
Manabe et al. (2015)	Phase III, Japanese	42	7	Imatinib + CT + HSCT	CR: 86%, MRD-: 11 patients, OS 4 years: 78.1% ± 6.5%. EFS 4 years: 54.1% ± 7.8%	Not evaluated	Moderate risk	Moderate
Biondi et al. (2018)	Phase III, open	160	1.5-17.9 (median not mentioned)	Imatinib + CT post-induction	EFS 4 years: 72.9% [95% CI, 56.1-84.1] (std) 53.5% [95% CI, 40.4-65.0] (HR)	Common infections, myelosuppression	Moderate risk	Moderate
Rives et al. (2011)	Phase III, multicenter	43	6.8	Imatinib + CT	EFS 3 years: 78.7% vs. 29.6% (p = 0.01)	Mucositis and nausea grade 3-4, elevated transaminases levels	Serious risk	Moderate
Brown et al. (2021a)	Phase III, two arms	127	170–179 days	Lestaurtinib + CT vs. only CT	EFS 3 years: no difference (p = 0.45)	Diarrhea more frequent with lestaurtinib	Serious risk	High
Rheingold et al. (2017)	Phase I	16	9	Temsirolimus + CT	4 CR, 3 PR	Neutropenic fever (67%) and infections (40%)	Moderate risk	Moderate
Proteasome inhibitor therapy								
Bertaina et al. (2017)	Phase III, monocentric	30	<15	Bortezomib + reinduction	CR/PR: 72.9%, OS: 24.4% [95% CI, 8.8-44.1]	Neuropathy (14.7%)	Moderate risk	Moderate
Messinger et al. (2012)	Phase II	22	12	Bortezomib + DEX	CR: 64% [95% CI, 41-83] OS 24 m: 41% ± 13%	3 deaths caused by sepsis	Moderate risk	Moderate
August et al. (2020)	Phase II	10	0.9–18.5 (median not mentioned)	Bortezomib + ALL-R3 scheme	5 CR	40% ≥Grade 3 AE, infections	Serious risk	Moderate
Iguchi et al. (2017)	Phase II	6	10–16	Bortezomib schemes A/B	Four of five patients with CR or CRi	1 death from pneumonitis	High risk	Moderate

CT: chemotherapy, DEX: dexamethasone, MRD: minimal residual disease, AE: adverse event, EFS: Event-free survival, y: years, m: months, CR: complete remission, CRi: Complete remission without platelet count recovery, std: standard, HR: high risk, PR: partial response, OS: overall survival, RoB2: Risk of bias 2, ROBINS-I: Risk Of Bias In Non-randomised Studies of Interventions, GRADE: grading of recommendations, Assessment, Development and Evaluation. HSCT: Hematopoietic stem cell transplantation. N: sample size. 95% CI: 95% Confidence Interval.

### 3.2 Proteasome inhibitors

Another therapeutic class with increasing evidence in pediatric B-ALL patients is proteasome inhibitors (PIs). These agents target the ubiquitin–proteasome pathway, a cellular mechanism essential for protein degradation and turnover, impacting key processes such as apoptosis, DNA repair, and antigen presentation (Kaplan, et al., 2017). The 26S proteasome, which is central to this pathway, has become a therapeutic target because its inhibition leads to disruption of the cell cycle, suppression of proliferation, and induction of apoptosis in malignant cells (Zhang, et al., 2022). Additionally, PIs are believed to exert their antitumor effects by modulating antiapoptotic proteins (e.g., Bcl-2 and Bcl-xL) and interfering with NF-κB signaling.

Bortezomib, the first-in-class PI approved for clinical use in 2004. Its mechanism centers on inhibiting the chymotrypsin-like activity of the 26S proteasome, promoting apoptosis in rapidly dividing cells. A phase II trial combining bortezomib with chemotherapy in relapsed/refractory B-ALL reported an overall response rate of 80% and a bone marrow response in 85% of patients (Messinger, et al., 2012). Similarly, a phase III reinduction study achieved complete remission in 22 out of 30 children, although the overall survival was 24.4% (Bertaina, et al., 2017). Other phase II trials reported response rates ranging from 55% to 80% in this setting (Iguchi, et al., 2017; August, et al., 2019).

A retrospective Mexican cohort that included 15 pediatric and young adult patients with B-ALL reported: a complete response was achieved in 60% of patients, 13% showed a partial response and no deaths were reported during treatment (Colunga-Pedraza, et al., 2020). These outcomes highlight the potential role of bortezomib as an adjunctive agent in resistant or relapsed patients.

Nonetheless, its safety profile must be carefully managed. Neurotoxicity, hematologic suppression, and gastrointestinal events have been consistently reported. In clinical trials, motor or sensory neuropathy occurs in approximately 15% of patients, whereas more than 40% of patients experience fluid and electrolyte disturbances (Bertaina et al., 2017). In the Mexican series, dose reduction was necessary in 60% of cases due to toxicity concerns, yet therapeutic efficacy was preserved. Mild to moderate peripheral neuropathy and gastrointestinal adverse events were the most reported adverse events, with no treatment-related deaths reported (Colunga-Pedraza, et al., 2020).

### 3.3 Immunological antineoplastic therapies

The immune system plays a pivotal role in the regulation of cancer, particularly in leukemias. Under normal physiological conditions, this system recognizes and eliminates tumor-specific antigens. However, cancer cells possess the capacity to evade this surveillance mechanism (Wyatt and Bram, 2019).

A comprehensive understanding of this process and its relationship with to B-cell ALL acute lymphoblastic leukemia has facilitated the development of a critical component of targeted therapies: immunological antineoplastics. These therapies harness the body’s intrinsic defense mechanisms to target leukemic cells through antibodies that recognize antigens expressed on blasts or by modifying immune cells, such as T lymphocytes, to become sensitized to leukemic cells.

Antineoplastic immunotherapy for the treatment of B-cell ALL in pediatric patients can be broadly categorized into three groups: targeted antibody therapy, antibody-drug conjugates and chimeric antigen receptor CAR-T cell therapy.

#### 3.3.1 Targeted antibody therapy

The humoral immune response, which is mediated by B lymphocytes, depends on the selection, activation, and clonal expansion of lymphocytes that express specific membrane-bound B-cell receptors (BCRs). This diversity of BCRs enables recognition of a vast array of antigens, and only those lymphocytes whose receptors bind a particular antigenic epitope undergo activation and differentiation into memory B cells or antibody-secreting plasma cells (Abbas, 2022).

**FIGURE 3:**
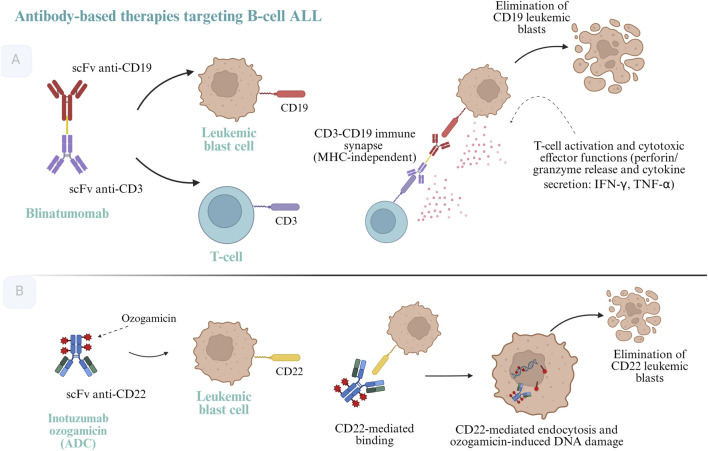
Antibody-based therapies in pediatric B-cell acute lymphoblastic leukemia. **(A)** Blinatumomab is a bispecific T-cell engager composed of two single-chain variable fragments (scFv) that simultaneously bind CD3 on T lymphocytes and CD19 on B-cell leukemic blasts. By physically linking T cells to CD19-positive target cells, blinatumomab induces the formation of an immunological synapse, leading to T-cell activation, cytotoxic effector function, and the elimination of CD19-positive leukemic blasts. **(B)** Inotuzumab ozogamicin is an antibody–drug conjugate (ADC) consisting of a humanized anti-CD22 monoclonal antibody conjugated to the cytotoxic agent ozogamicin. Upon binding to CD22 expressed on B-cell leukemic blasts, the ADC–antigen complex is internalized, resulting in intracellular release of ozogamicin, induction of DNA double-strand breaks, and apoptotic cell death, ultimately leading to the elimination of CD22-positive leukemic blasts. Created in BioRender. CORREA, V. (2026) https://BioRender.com/nmbkbkx.

Antibodies are soluble forms of BCRs produced by plasma cells, retain antigen specificity and mediate diverse effector mechanisms, such as neutralization, opsonization, complement activation, and engagement of cytotoxic immune cells, such as macrophages and NK cells (Abbas, 2022). Targeted antibody therapies harness this mechanism to eliminate malignant cells by directing the immune response toward tumor-specific antigens expressed by leukemic blasts, as shown in Figure 3.

##### 3.3.1.1 Bispecific antibodies

Bispecific T-cell engager antibodies are engineered molecules capable of simultaneously binding two distinct antigens, typically a tumor-associated antigen and a T-cell–specific marker, thereby facilitating direct cytotoxic synapse formation between immune effector cells and tumor cells (Brivio, et al., 2022).

Blinatumomab is a bispecific antibody that targets CD19 on B cells and CD3 on T cells, triggering T-cell activation and subsequent lysis of malignant B-lineage cells (Van der Sluis et al., 2023; Farma, 2023). Given its short half-life (∼3 h), it requires continuous intravenous infusion to maintain therapeutic plasma concentrations (Rivas, 2016).

CD19 is expressed at most stages of B cell development and is consistently present in B-cell acute lymphoblastic leukemia, making it an effective target. Clinical trials have demonstrated the superiority of blinatumomab over chemotherapy in specific settings: in children with first relapsed B-ALL, blinatumomab improved 2-year event-free survival (54.5% vs. 39%) and overall survival (71.3% vs. 58.4%) (Brown, et al., 2021b). Additionally, it resulted in greater MRD clearance as a third-line consolidation therapy (90% vs. 54%) (Locatelli, et al., 2021). A 2024 pilot study also suggested that blinatumomab could maintain MRD negativity during early treatment phases, supporting its use as a first-line agent (Mikhailova, et al., 2024).

Blinatumomab demonstrates a more favorable safety profile compared to chemotherapy. Regarding safety, a lower frequency of various adverse effects has been reported in patients receiving blinatumomab compared to patients receiving chemotherapy: in serious infections (24.1% vs. 43.1%), febrile neutropenia (5% vs. 58%), and sepsis (2% vs. 27%), adverse effects greater than or equal to grade 3 (57.4% vs. 82.4%) (Brown, et al., 2021b; Locatelli, et al., 2021). Nonetheless, immune-related adverse events such as cytokine release syndrome and neurotoxicity have been reported, although they are generally manageable with appropriate monitoring and dose adjustment.

#### 3.3.2 Antibody–drug conjugates (ADCs)

While monoclonal antibodies represent the first generation of targeted cancer therapies that selectively bind tumor-specific antigens, their clinical efficacy as monotherapies has often been limited. To enhance therapeutic potency, a new class of immunoconjugates -antibody–drug conjugates (ADCs)- was developed. These molecules couple a monoclonal antibody with a cytotoxic agent via a stable chemical linker, allowing the precise delivery of chemotherapy directly to malignant cells. In this design, the antibody functions as a vector that targets leukemia cells, while the attached drug exerts its cytotoxic effect intracellularly (Fu et al., 2022).

The first ADC approved by the FDA was gemtuzumab ozogamicin in 2000 for the treatment of acute myeloid leukemia, marking the beginning of a new therapeutic era (U.S. Food and Drug Administration, 2000). Since then, multiple ADCs have been developed across different hematologic and solid malignancies. The structure of an ADC generally includes three components: a monoclonal antibody targeting a tumor antigen, a potent cytotoxic payload, and a linker that maintains stability in circulation but permits intracellular release (Fu et al., 2022).

Inotuzumab ozogamicin (InO) is a representative ADC used in B-cell precursor acute lymphoblastic leukemia. It consists of a humanized IgG4 monoclonal antibody directed against CD22, a surface marker present in >90% of B-ALL blasts, conjugated to calicheamicin, a highly potent DNA-cleaving agent. The linkage is achieved through a hydrazone bond that is cleaved in the acidic lysosomal environment following receptor-mediated internalization (LiverTox, 2024; Fu et al., 2022). Once internalized, the cytotoxic agent is released and induces double-strand DNA breaks, triggering apoptosis in leukemic cells (LiverTox, 2024).

InO was approved by the FDA in 2017 for use in adults with refractory B-ALL leukemia. More recently, in March 2024, its indications were expanded to include pediatric patients over 1 year of age in the United States, followed by regulatory approval in Japan (Dhillon, 2024).

Studies in pediatric patients are still in early stages, reporting an overall response rate after the first cycle of 80% (Brivio, 2021), negativity in MRD in 81.8% of responders, and overall survival at 12 months of 56.3% (Pennesi et al., 2022). The most frequent adverse effects were fever, cytopenias, and transient elevations in liver enzymes (Brivio, 2021; Pennesi et al., 2022). The risk of veno-occlusive disease, particularly in patients undergoing subsequent hematopoietic stem cell transplantation, requires careful monitoring and has been addressed through risk-adapted dosing schedules and patient selection criteria. Overall, InO offers a potent and targeted therapeutic option for high-risk pediatric B-ALL, especially in relapsed or refractory settings, balancing efficacy with an acceptable safety profile when used under appropriate clinical supervision.

#### 3.3.3 CAR-T cell therapy

One of the most transformative advances in immuno-oncology has been the development of chimeric antigen receptor (CAR) T-cell therapy, which involves engineering a patient’s own T lymphocytes to recognize tumor antigens independently of the major histocompatibility complex (MHC) (Dai, et al., 2016). This bypasses one of the key immune evasion strategies used by cancer cells: the downregulation of MHC molecules and costimulatory ligands, which otherwise limits cytotoxic T-cell activation (Gardner, 2017).

CARs are synthetic receptors that combine antigen-specificity derived from antibodies with intracellular T-cell activation domains.

**FIGURE 4:**
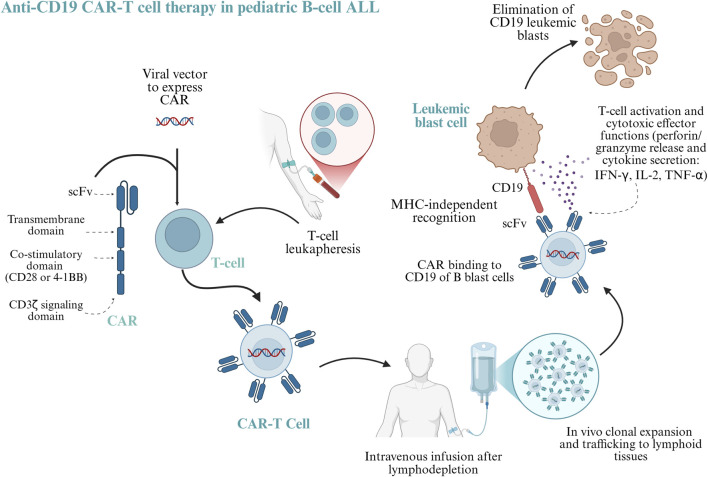
Anti-CD19 CAR-T cell therapy in pediatric B-cell acute lymphoblastic leukemia. Autologous T cells, obtained by leukapheresis and genetically modified using viral vectors to express a chimeric antigen receptor (CAR) composed of an extracellular single-chain variable fragment (scFv) specific for CD19, a transmembrane region, and intracellular signaling domains, including CD3ζ and a co-stimulatory domain (CD28 or 4-1BB). Following lymphodepleting conditioning and intravenous infusion, CAR-T cells undergo *in vivo* clonal expansion and traffic to lymphoid tissues and the bone marrow. CAR-mediated recognition of CD19 occurs in a major histocompatibility complex (MHC)-independent manner, leading to T-cell activation, proliferation, and cytotoxic effector functions. These include perforin- and granzyme-mediated target cell lysis and the secretion of proinflammatory cytokines such (IFN-γ, IL-2 and TNF-α), which eliminate leukemic cells. The persistence and memory of CAR-T cells contribute to sustained antitumor surveillance. Created in BioRender. CORREA, V. (2026) https://BioRender.com/nmbkbkx.

Their architecture includes an extracellular antigen recognition region, composed of a single-stranded variable fragment, a fusion protein of the variable regions of heavy and light immunoglobulins connected by a short, flexible peptide bond; a hinge or spacer that optimizes epitope accessibility; a transmembrane segment; and intracellular signaling motifs that initiate T-cell activation, such as CD3ζ, as well as costimulatory motifs, such as CD28 found in KTE-X19 CAR-T cells, or 4-1BB found in Tisacel, these factors improves the persistence of CAR-T cells in the patient and produces better treatment outcomes (Uscanga-Palomeque et al., 2023). By providing direct recognition of surface antigens, independent of major histocompatibility complex presentation, CARs effectively replace the native T-cell receptor (Figure 4).

In 2017, the FDA approved the first CAR-T cell therapy: Tisagenlecleucel, targets CD19 for relapsed or refractory B-ALL in children and relapsed or refractory large B-cell lymphoma in adults (Uscanga-Palomeque et al., 2023). In recent years, another therapeutic target in CD22 has been studied for CD19-negative relapse.

The manufacturing process involves harvesting T cells through apheresis, genetically modifying them to express the CAR construct, and reinfusing them into the patient after lymphodepleting chemotherapy (Patel, et al., 2023).

Among the CAR-T-cell products currently approved, Tisagenlecleucel was the first to receive FDA approval, granted in 2017 for use in pediatric and young adult patients with refractory or multiple relapsed B-cell acute lymphoblastic leukemia (Schultz, 2020). Clinical trials have reported remarkable response rates, with survival rates exceeding 90% in some cohorts; however, these results are from early-stage studies, where the maximum follow-up time was only 18 months (Maude et al., 2018; Pan et al., 2023). CART-T-cell use is accompanied by a distinct toxicity profile, the most relevant being cytokine release syndrome and neurotoxicity, which can range from mild to life-threatening. Current pediatric studies with tisagenlecleucel are summarized in Table 2.

**TABLE 2:** Outcomes of clinical trials in pediatric patients with B-cell acute lymphoblastic leukemia treated with immune-mediated antineoplastic therapy.

Author (year)	Study	N	Median age (y)	Intervention	Efficacy	Safety	RoB2/ROBINS-I	GRADE
Bispecific antibody therapy								
Brown et al. (2021a)	Phase III	208	9	Blinatumomab vs. CT	EFS 2 years: 54.4% vs. 39% [95% CI, 0.47-1.03] OS 2 years: 71.3% vs. 58.4% [95% CI, 0.39-0.98]	Any AE ≥ Grade 3: 83% vs. 90%	Some concerns	Moderate
Locatelli et al. (2021)	Phase III	108	5	Blinatumomab vs. CT	MRD-: 90% vs. 54% [95% CI, 15.6-52.5], EFS: 37% vs. 23% [95% CI, 0.18-0.61]	Any AE ≥ Grade 3: 57,4% vs. 82,4%. Serious EA: neurologic symptoms 3.7% and seizure 3.7% vs. febrile neutropenia 17.6%	Some concerns	High
Stackelberg (2016)	Phase I/II	93	6–10.5 (median not mentioned)	Blinatumomab (2 cycles)	CR: 32% [95% CI, 19-48]	6 deaths, neurological AE ≥ Grade 3: 4%	Moderate risk	Moderate
Van der Sluis et al. (2023)	Phase II	30	<1	Blinatumomab + Interfant-06	MRD-: 93%, OS 2 years: 93.3% [95% CI, 75.9-98.3]	No neurological events. Non hematological AE ≥ Grade 3: 30%	Serious risk	Moderate
Mikhailova et al. (2024)	Pilot study	117	4.1	Blinatumomab after induction	MRD-: 99.2%. [95% CI not reported]	Not evaluated	Moderate risk	Low
Horibe et al. (2020)	Phase Ib, open	9	11	Blinatumomab as induction	M1 remission rate: 44% [95% CI not reported]	Any AE ≥ Grade 3: 89%	Moderate risk	Very low
Antibody-drug conjugate								
Pennesi et al. (2022), Pennesi et al. (2024)	Phase I/II	28	7.5	InO 1.8 mg/m^2^ cycle	CR: 81.5% [95% CI 61.9-93.7], MRD-: 81.8% EFS 1 year: 36.7% [95% CI 22.2-60.4] OS: 55.1% [95% CI 39.1-77.7]	Neutropenia grade 3-4: 93%, Alanine aminotransferase increase grade >3: 33.3%. Aspartate aminotransferase increase grade >3: 50%, anemia grade >3: 63.3%	Serious risk	Very low
Brivio (2021)	Phase I	25	11	InO scaling	CR: 80 [95% CI 59-93]–85% [95% CI 55-98] MRD-: 84% [95% CI 60-97] OS 12 m: 40% [95% CI 25-66]	4 deaths (progression, sepsis) any AE ≥ Grade 3: 92%	Moderate risk	Low
CAR-T cell therapy								
Maude et al. (2018)	Phase II global	75	11	Tisagenlecleucel	CR: 81%, OS 12 m: 76% [95% CI 63-86], EFS 73% [95% CI 60-82], OS: 90% [95% CI 81-95]	Cytokine release 77%, AE grade 3-4: 73%. Neurological events in 40%	Moderate risk	Moderate
Pan et al. (2023)	Phase II, monocentric	81	8	CD19-targeted CAR T cells 2.7 x 10^6^/kg, CD22-targeted CAR T cells 2.2 x 10^6^/kg	MRD or CRi: 60/62 and 79/81 patients [95% CI 89-100], OS 18 m: 96%, EFS 18 m: 79% [95% CI 66-91]	Cytopenias 79% Grade 3-4	Serious risk	Moderate

CT: chemotherapy, InO: inotuzumab ozogamicin, MRD: minimal residual disease, AE: adverse event, EFS: Event-free survival, y: years, m: months, CR: complete remission, CRi: Complete remission without platelet count recovery, OS: overall survival, RoB2: Risk of bias 2, ROBINS-I: Risk Of Bias In Non-randomised Studies of Interventions, GRADE: grading of recommendations, Assessment, Development and Evaluation. N: sample size. 95% CI: 95% Confidence Interval.

### 3.4 Cost-benefit of targeted therapies in B-ALL pediatric population

Targeted immunotherapies have significantly improved outcomes in pediatric patients with B-cell acute lymphoblastic leukemia; however, their high costs pose a challenge for healthcare systems.

The analysis by Díaz (2024) is particularly relevant for Latin America, where Blinatumomab has a 99% probability of being cost-effective compared to conventional chemotherapy. Although its total cost is higher (≈65,110 USD vs. ≈45,474 USD), this is offset by a gain of 5.11 life years and reduced costs associated with adverse events and hospitalization.

On the other hand, therapies such as Tisagenlecleucel (CAR-T cells) can exceed 475,000 dollars. Nonetheless, they offer substantial benefits: around 40% of treated children achieve long-term remission, compared to 10% with conventional therapies (Whittington, 2018). In high-income countries, their cost-effectiveness is estimated at approximately $46,000 per quality-adjusted life year (QALY), a threshold considered acceptable (Borgert, 2021).

However, extrapolating these economic models to low- and middle-income countries requires caution. Reference thresholds (e.g., ≈€50,000/QALY in France) often exceed the capacity of Latin American health systems, where the WHO recommends thresholds based on *per capita* Gross Domestic Product (1–3 times), which are significantly lower.

Despite the above, evidence suggests that the high initial cost of therapies such as CAR-T or Blinatumomab could be offset in these contexts by a reduction in hospital burden. By presenting more manageable toxicity profiles, these therapies could alleviate critical bottlenecks, such as the shortage of intensive care beds and the management of complications like febrile neutropenia, thereby offering an operational efficiency that models from developed countries do not always prioritize.

It should be noted that the available economic evidence has limitations. Most studies come from high-income countries, and the costs of advanced therapies vary drastically depending on national reimbursement agreements. Research using models based on local costs is needed to validate the long-term sustainability of these therapies in Latin America.

### 3.5 Efficacy

To facilitate interpretation of the heterogeneous evidence, the narrative findings were complemented with a synthesis of ranges and trend patterns across targeted therapies. Response rates among protein kinase inhibitors varied widely, with complete remission (CR) ranging from 59% to 98%, while event-free survival spanned 36%–78% across studies. Immunotherapies demonstrated more consistent results: CAR-T therapies achieved CR rates of 75%–93% and 12-month overall survival of 70%–96%, whereas blinatumomab showed superior minimal residual disease clearance compared with chemotherapy (90% vs. 54%). In contrast, FLT3 inhibitors, mTOR inhibitors, and CDK4/6 inhibitors exhibited inconsistent efficacy signals, highlighting substantial variation in the strength of evidence.

### 3.6 Safety

Across all therapies, reporting of safety outcomes remained heterogeneous, therefore Common Terminology Criteria for Adverse Events criteria were applied to improve comparability where possible. Tyrosine kinase inhibitors consistently demonstrated high rates of grade 3–4 hematologic toxicities, primarily neutropenia, thrombocytopenia, and anemia, often exceeding 50%, with non-hematologic adverse events, such as gastrointestinal or hepatic events, occurring less frequently. Proteasome inhibitors showed a distinctive pattern characterized by peripheral neuropathy (10%–15%), electrolyte disturbances (>40%), and infections, with hematologic suppression being common. Antibody-based therapies exhibited more favorable profiles: blinatumomab had a lower frequency of severe infections and febrile neutropenia compared with chemotherapy, while inotuzumab ozogamicin carried a specific risk of hepatotoxicity and veno-occlusive disease, particularly in post-transplant settings. CAR-T cell therapies presented unique immune-mediated toxicities, primarily cytokine release syndrome and acquired immunodeficiency syndrome, with grade ≥3 neurotoxicity in 20%–28% of cases. Direct comparisons with chemotherapy showed that, except for CAR-T cells, most targeted agents reduced the rates of systemic complications and serious infections, thus improving overall tolerability.

### 3.7 Quality of life of targeted therapies in pediatric population with LLA-B

In the evaluation of health-related quality of life in pediatric oncology patients, scales such as the Pediatric Quality of Life Inventory 3.0 are used (Ocak et al., 2021). However, we did not find reports where quality of life scales are applied and the results of these assessments are reported, particularly in patients who have received some type of targeted therapy. Therefore, this represents an important research gap that should be addressed in future studies to assess the quality of life of patients with ALL.

We conducted a targeted search and found only one article, published in 2012, on adults with acute myeloblastic leukemia. This article aimed to compare the important aspects of quality of life as perceived by patients versus those perceived by healthcare professionals. The elements on which both groups agreed were: fatigue, muscle cramps, swelling, concerns and uncertainty about future health status, and the importance of social support in coping with the disease (Efficace et al., 2012).

## 4 Advantages and disadvantages of targeted therapies

According to the results reported by the studies included in this review and an analysis of the literature consulted during the preparation of this review, we point out that targeted therapies have advantages and disadvantages (Table 3) and that their use should be balanced in consideration of this information, with the goal of achieving better outcomes in the health status of the pediatric oncology patients.

**TABLE 3:** Advantage and limitation of targeted therapies.

Advantage	Limitation
Labeling of neoplastic cells for destruction	Few studies testing targeted therapies as monotherapy
Specifically targets malignant cells	Few studies in pediatric population
Improves event-free survival	Only seven phase 3 studies found
Improves overall survival in relapsed patients	No reports evaluating quality of life in patients treated with these therapies
Can be combined with conventional therapy	No effectiveness or safety studies in Latin American pediatric population

## 5 Challenges

Among the therapies reviewed, CAR-T cells targeting CD19 have demonstrated the highest clinical remission rates, exceeding 80% in pediatric and young adult patients with relapsed or refractory ALL; however, it will be important to monitor the results of future studies with longer follow-up periods and larger sample sizes. The integration of targeted therapies into the treatment paradigm for pediatric B-cell ALL faces several significant challenges. These challenges span evidence gaps, safety considerations, accessibility, and biological limitations.

First, a critical evidence gap exists, as most clinical trials have focused on relapsed or refractory disease, leaving a lack of robust data for their use in frontline treatment. Furthermore, the absence of head-to-head comparisons between different targeted therapies hinders the identification of optimal treatment sequences or combinations based on efficacy and tolerability.

Persistent toxicity remains a concern, requiring structural modifications to reduce adverse effects and optimization of administration routes. These efforts must be coupled with careful assessment of their impact on quality of life, a particularly critical outcome in pediatric oncology. Finally, high costs and limited access to these advanced therapies, especially within Latin American populations, represent important barriers to their implementation.

Beyond these clinical and structural limitations, biological resistance represents a central challenge for CD19-directed therapies. One of the main challenges facing CD19-directed targeted immunotherapies, including CAR-T cell therapy and bispecific T-cell engagers such as blinatumomab, is the emergence of resistance mechanisms associated with their use. To address this issue, a targeted search for resistance mechanisms was conducted. In the study by Orlando et al., 2018 resistance to CD19-directed CAR-T cell therapy was primarily associated with antigenic escape mediated by acquired genetic alterations (Orlando et al., 2018). In patients who developed CD19-negative relapse, loss-of-function mutations in the CD19 gene were identified at the time of relapse but were absent in baseline samples. These alterations generated truncated proteins lacking a functional transmembrane domain, resulting in complete loss of CD19 surface expression and impaired recognition by CD19-targeted immune effectors, including CAR-T cells and blinatumomab (Orlando et al., 2018; Jacoby and Shah, 2019). In most cases, concomitant loss of heterozygosity at the CD19 locus was observed, supporting a biallelic inactivation model as a central mechanism of resistance (Orlando, et al., 2018). Subsequent studies extended this paradigm by demonstrating that reduced CD19 surface density, even in the absence of complete antigen loss, is sufficient to confer resistance, as observed in CD19-low relapses following CD19-directed immunotherapies (Spiegel, et al., 2021; Jacoby and Shah, 2019). Collectively, these findings indicate that both genetic inactivation and quantitative modulation of the target antigen represent complementary mechanisms of resistance (Orlando, et al., 2018; Spiegel, et al., 2021). In addition, limited persistence and functional exhaustion of engineered T-cell–based therapies have been described as important contributors to treatment failure, as these processes impair cellular expansion and sustained antitumor cytotoxic activity, particularly under conditions of continuous antigenic stimulation (Jacoby and Shah, 2019; Lesch et al., 2020).

Taken together, these challenges highlight key areas for future development. Future efforts must be strategically directed, research priorities should include expanding clinical trials into earlier lines of therapy, conducting comparative effectiveness studies, and enhancing safety profiles.

## 6 Conclusion

Based on the available evidence, targeted therapies may differ from conventional chemotherapy in terms of efficacy, safety, and quality-of-life outcomes in pediatric patients with B-cell acute lymphoblastic leukemia. However, according to GRADE assessments, the certainty of evidence supporting these comparisons is low to very low, primarily due to the limited number of direct comparative studies, imprecision, heterogeneity, and a moderate to high risk of bias identified using the RoB 2 tool and a moderate risk of bias using ROBINS-I.

While CD19-directed CAR-T cell therapy, blinatumomab, inotuzumab ozogamicin, and tyrosine kinase inhibitors in Philadelphia chromosome–positive ALL show effective activity, particularly in relapsed or minimal residual disease–positive settings, current evidence does not allow definitive conclusions regarding their superiority over standard chemotherapy across treatment lines.

Well-designed, adequately powered randomized controlled trials with standardized comparators, robust quality-of-life measures, and longer follow-up are required to improve the certainty of evidence and to define the optimal role of targeted therapies, particularly in frontline treatment. Future research should also address equity in access to these therapies to ensure that potential benefits are realized across diverse pediatric populations.

## 7 Study limitations

During the review process, several limitations were identified: heterogeneity of studies, variability in clinical trial designs, populations studied, and interventions evaluated complicate direct comparisons of results. The heterogeneity in treatment (monotherapy or combinations) reflects the current clinical reality in pediatric ALL.

Risk of bias: The included studies presented a heterogeneous risk of bias, primarily determined by their design. Randomized controlled trials, assessed using RoB 2, generally showed a risk of bias with some concern, the main limitation being the lack of blinding. However, they had adequate randomization, intention-to-treat analysis, and objective outcomes, which reduced the impact of bias. In contrast, most non-randomized studies, assessed using ROBINS-I, presented a moderate overall risk of bias, mainly due to residual confounding and selection bias stemming from the absence of comparators and clinical heterogeneity of the included populations. This bias predominantly leaned toward overestimating the benefit of the interventions, while the domains related to the definition of the intervention, the measurement of outcomes, and the handling of missing data consistently showed a low risk of bias. These aspects were considered in the interpretation of the results.

Quality of evidence: when comparing the strength of evidence among targeted therapies, clear differences emerge. The most robust data come from bispecific antibodies such as blinatumomab, supported by multiple phase II–III trials with consistent improvements in remission, MRD clearance, and survival. Inotuzumab ozogamicin also shows substantial activity, although supported by smaller cohorts. Conversely, FLT3 inhibitors, mTOR inhibitors, and CDK4/6 inhibitors are characterized by early-phase studies with small samples and modest or inconsistent responses, indicating weaker or preliminary evidence. Distinguishing these gradients of certainty is essential for guiding clinical decision-making and prioritizing future research efforts.

Scarcity of data on quality of life: despite being a key outcome in the pediatric population, few studies have reported results related to quality of life. These limitations highlight the need for more high-quality research with robust designs to strengthen the available evidence.

## Data Availability

The original contributions presented in the study are included in the article/Supplementary Material, further inquiries can be directed to the corresponding author.
