# Supplementary figures and images for: Targeted therapies in pediatric B-Cell acute lymphoblastic leukemia: mechanisms, efficacy, and future directions
Source: Front Pharmacol. 2026 Jan 28;17:1654783. doi: 10.3389/fphar.2026.1654783 (PMC12891086; doi:10.3389/fphar.2026.1654783)

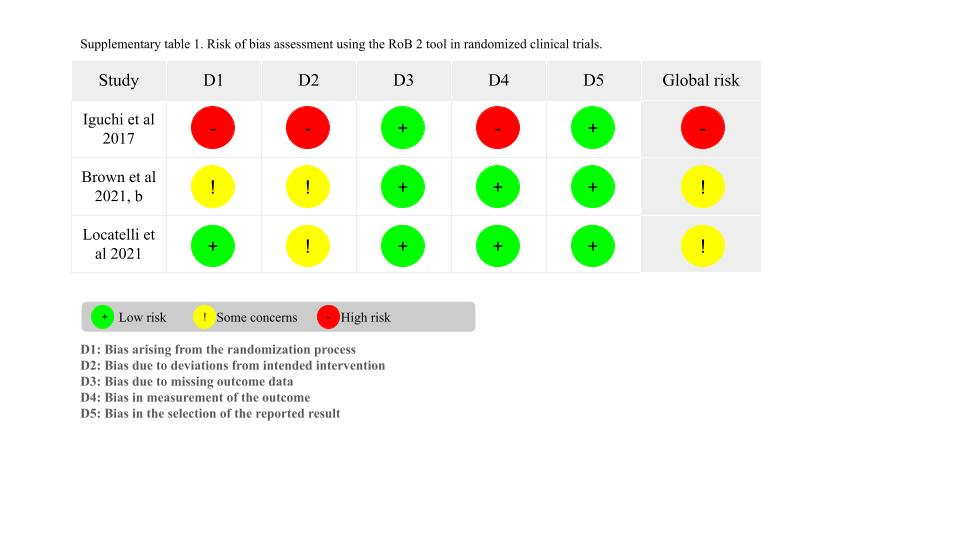

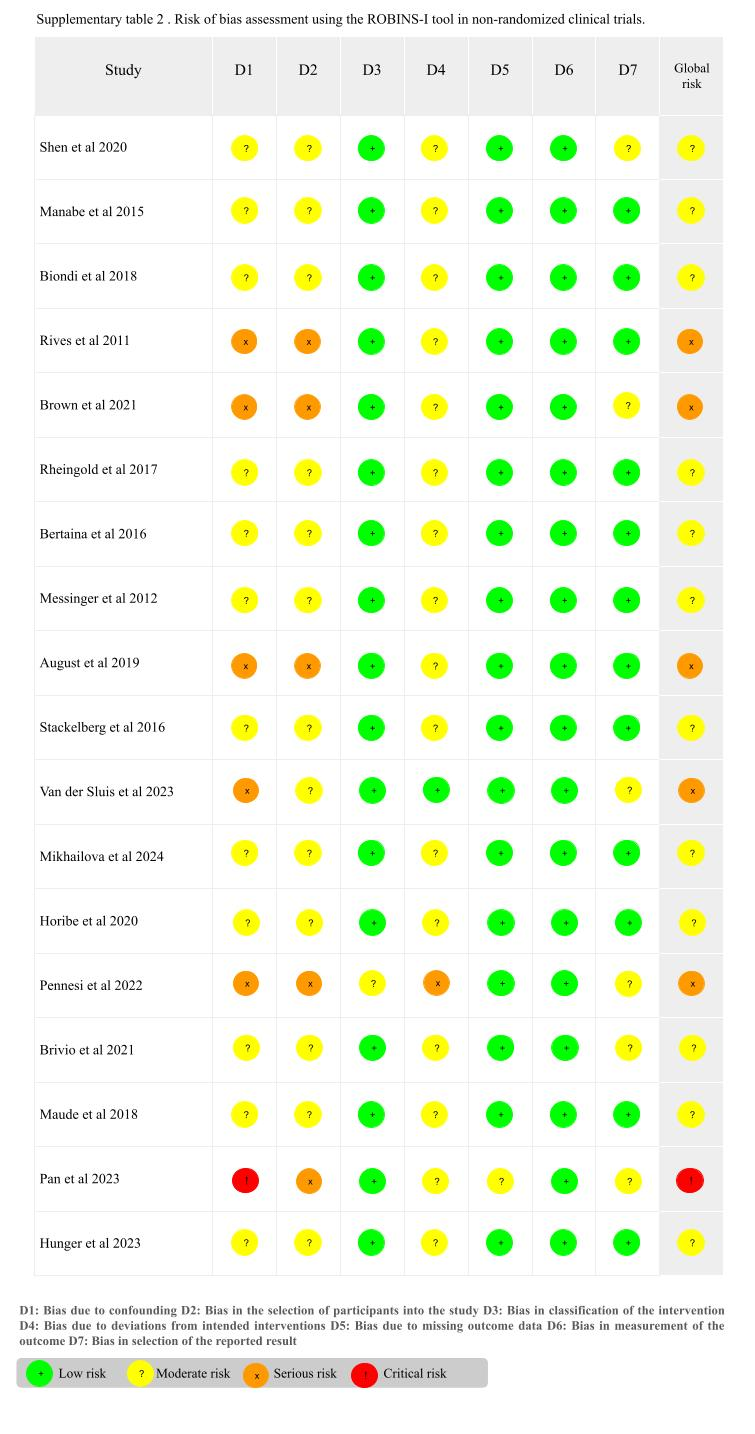

Supplement: Supplementary file 1 [file Supplementaryfile1.docx]
